# Post-translational Modification of LipL32 during *Leptospira interrogans* Infection

**DOI:** 10.1371/journal.pntd.0003280

**Published:** 2014-10-30

**Authors:** Timothy D. Witchell, Azad Eshghi, Jarlath E. Nally, Rebecca Hof, Martin J. Boulanger, Elsio A. Wunder, Albert I. Ko, David A. Haake, Caroline E. Cameron

**Affiliations:** 1 Department of Biochemistry and Microbiology, University of Victoria, Victoria, British Columbia, Canada; 2 School of Veterinary Medicine, University College Dublin, Belfield, Dublin, Ireland; 3 Department of Epidemiology of Microbial Disease, Yale School of Public Health, New Haven, Connecticut, United States of America; 4 Gonçalo Moniz Research Center, Oswaldo Cruz Foundation, Brazilian Ministry of Health, Salvador, Brazil; 5 Division of Infectious Diseases, Veterans Affairs Greater Los Angeles Healthcare System, Los Angeles, California, United States of America; 6 Department of Medicine, David Geffen School of Medicine at University of California Los Angeles, Los Angeles, California, United States of America; University of Tennessee, United States of America

## Abstract

**Background:**

Leptospirosis, a re-emerging disease of global importance caused by pathogenic *Leptospira* spp., is considered the world's most widespread zoonotic disease. Rats serve as asymptomatic carriers of pathogenic *Leptospira* and are critical for disease spread. In such reservoir hosts, leptospires colonize the kidney, are shed in the urine, persist in fresh water and gain access to a new mammalian host through breaches in the skin.

**Methodology/Principal Findings:**

Previous studies have provided evidence for post-translational modification (PTM) of leptospiral proteins. In the current study, we used proteomic analyses to determine the presence of PTMs on the highly abundant leptospiral protein, LipL32, from rat urine-isolated *L. interrogans* serovar Copenhageni compared to *in vitro*-grown organisms. We observed either acetylation or tri-methylation of lysine residues within multiple LipL32 peptides, including peptides corresponding to regions of LipL32 previously identified as epitopes. Intriguingly, the PTMs were unique to the LipL32 peptides originating from *in vivo* relative to *in vitro* grown leptospires. The identity of each modified lysine residue was confirmed by fragmentation pattern analysis of the peptide mass spectra. A synthetic peptide containing an identified tri-methylated lysine, which corresponds to a previously identified LipL32 epitope, demonstrated significantly reduced immunoreactivity with serum collected from leptospirosis patients compared to the peptide version lacking the tri-methylation. Further, a subset of the identified PTMs are in close proximity to the established calcium-binding and putative collagen-binding sites that have been identified within LipL32.

**Conclusions/Significance:**

The exclusive detection of PTMs on lysine residues within LipL32 from *in vivo*-isolated *L. interrogans* implies that infection-generated modification of leptospiral proteins may have a biologically relevant function during the course of infection. Although definitive determination of the role of these PTMs must await further investigations, the reduced immune recognition of a modified LipL32 epitope suggests the intriguing possibility that LipL32 modification represents a novel mechanism of immune evasion within *Leptospira*.

## Author Summary

Leptospirosis, caused by pathogenic *Leptospira* spp., constitutes an increasing global public health threat. Humans are accidental hosts, and acquire the disease primarily from contact with water sources that have been contaminated with urine from infected animals. Rats are asymptomatic carriers of infection and are critical for disease transmission to humans, particularly in urban slum environments. In this study, investigation of *Leptospira* directly isolated from the urine of infected rats showed acetylation or tri-methylation of the highly abundant leptospiral lipoprotein, LipL32. In comparison, *Leptospira* grown in culture did not result in any LipL32 lysine modifications. A synthetic peptide derived from LipL32 that incorporated a tri-methylated lysine modification exhibited less reactivity with serum from leptospirosis patients compared to an unmodified version of the peptide, suggesting LipL32 modifications may alter protein recognition by the immune response. This study reports, for the first time, modification of a *Leptospira* protein during infection, and suggests these modifications may have a functional consequence that contributes to bacterial persistence during infection.

## Introduction

Pathogenic *Leptospira* spp. are the causative agents of leptospirosis, which is considered to be the world's most widespread zoonotic disease [1]–[4]. Recent data shows the incidence of leptospirosis is increasing, with outbreaks frequently occurring within urban slum settings [5]–[8], an environment in which approximately 31.6% of the world's total urban population resides [9]. Therefore, leptospirosis represents a significant public health threat in these communities.

Mammalian hosts that are chronically infected with *Leptospira* contain the spirochetes within the renal tubules of the kidney. Dissemination of pathogenic *Leptospira* occurs largely via urine excreted from these infected hosts, with bacterial acquisition by new hosts occurring through cuts/abrasions or mucous membranes as a consequence of direct exposure to infectious urine or urine-contaminated water [1]. One of the most significant reservoir hosts of *Leptospira* is the brown rat, *Rattus norvegicus* [10], which is critical for the spread of leptospirosis among urban slum settings. Rats can shed *Leptospira* in urine at high densities (10^4^ to 10^7^ cells/ml) over a prolonged period of time due to persistent renal colonization, and without apparent detrimental effects on the health of the rat [11], [12]. Within humans, clinical symptoms associated with leptospirosis range from mild illness including fever, chills, headache and myalgia to serious sequelae including hepatic, renal or pulmonary disorders. Severe cases of leptospirosis, which frequently display renal involvement, are reported to be diagnosed in 5–10% of patients, with mortality rates among these patients estimated at 5–40% [13], [14]. Asymptomatic renal colonization and excretion of *Leptospira* within the urine of patients for weeks and, in rare instances, months have been reported [2], [15], [16].

Although the underlying mechanisms by which pathogenic *Leptospira* spp. are able to persist within mammalian hosts are incompletely understood, we have put forward the hypothesis that post-translational incorporation of methyl groups in leptospiral outer membrane proteins (OMPs) may enhance bacterial survival by modifying proteins and their respective functional interactions within the host. In support of this we have previously reported the addition of *O*-methyl esters to selected glutamic acid residues within the leptospiral protein OmpL32 [17], and Cao *et al.* have detected mono-, di- and tri-methylations on proteins from *L. interrogans* serovar Lai, including the major leptospiral lipoprotein LipL32 [18]. Also in support of this hypothesis is the observation in the Gram-negative bacterium *Rickettsia prowazekii* that methylation of OmpB alters the immunogenicity of this outer membrane protein [19]. Further, OmpB proteins from the virulent *Rickettsia* Breinl and Evir strains have been shown to be more extensively methylated, and more protective, than OmpB from the avirulent Madrid E strain [20]–[24].

In the current study we investigate the presence of PTMs, including methylations, occurring within the major leptospiral lipoprotein LipL32. Since LipL32 is found only within pathogenic *Leptospira* serovars and constitutes the most abundant protein within the cell (approximately 38,000 copies per cell) [25], the incorporation of PTMs within LipL32 could have significant implications for the currently elusive function of this protein. Herein we analyzed the extent of LipL32 PTMs observed in leptospires directly isolated from a rat chronic infection model in comparison with *in vitro*-cultured *Leptospira*. Through these analyses we show that PTMs, composed of either tri-methylations or acetylations, occur solely on lysine residues from *in vivo*-derived LipL32. Four PTMs occurred in regions of LipL32 previously identified as B cell epitopes, and one PTM decreased epitope recognition by human leptospirosis serum. This study provides the first *in vivo* proteomic investigation of post-translational modifications of a major leptospiral lipoprotein and provides insight into the potential functional consequences of such modifications.

## Methods

### Ethics statement

Samples were obtained from patients with confirmed leptospirosis [5] and healthy control individuals [7] who were residents of the city of Salvador, Brazil and provided written informed consent. The study protocol was approved by the institutional review boards of Yale University and Oswaldo Cruz Foundation. Leptospires shed in rat urine were collected using a protocol approved by the University College Dublin Animal Research Ethics committee, approval P-42-05, and licensed by the Department of Health and Children, Ireland, license number B100/3682.

### Bacteria

*Leptospira interrogans* serovar Copenhageni strain RJ16441, a human clinical isolate [26] was passaged through guinea pigs to maintain virulence as previously described [27]. *In vitro* cultivated *Leptospira* (IVCL) samples were cultured at 30°C in Ellinghausen-McCullough-Johnson-Harris liquid medium (EMJH; Becton Dickinson, Oxford, England) [28], [29] supplemented with 6% rabbit serum (Sigma-Aldrich, Arklow, Ireland). Bacteria were enumerated by dark-field microscopy, harvested by centrifugation at 12,000× *g* for 10 min at 4°C when cultures reached a density of 1×10^8^ leptospires/ml, and bacterial pellets were washed three times with 10 mM Tris pH 7.4/1 mM EDTA and stored at −20°C. Rat urine isolated *Leptospira* (RUIL) samples were prepared as previously described [12]. Briefly, 6 six-week old male *Rattus norvegicus* Wistar strain animals (150–190 g, Charles River Laboratories, UK) were infected by intraperitoneal injection with 5×10^7^ low passage *in vitro* cultivated *Leptospira*. Upon detection of infection, rats were placed into metabolic cages and urine was collected twice weekly for six weeks. Urine samples were centrifuged at 12,000× *g* for 10 min at 4°C and pellets were washed and stored at −20°C as outlined above.

### Sample preparation and two dimensional gel electrophoresis

IVCL pellets (8×10^8^ cells) and RUIL pellets (a total of 12 pellets collected from six rats) were solubilized by overnight incubation at room temperature in a total volume of either 80 µL (IVCL) or 240 µL (RUIL) rehydration buffer (7M urea, 2M thiourea and 1% ASB-14). The protein concentration of the IVCL and RUIL samples was determined using the RC/DC protein assay kit (Bio-Rad, Hertfordshire, UK). A total of 100 µg of protein from the IVCL samples and 400 µg of protein from the RUIL samples was used to rehydrate 18 cm 4–7pH IPG strips overnight, and proteins were separated by two dimensional gel electrophoresis (2DGE) and stained with SYPRO Ruby as previously described [30], [31]. A higher amount of protein was used in the RUIL samples to compensate for the presence of rat urine proteins. Protein spots corresponding to LipL32 were identified by comparison with published data [32], [33] and the same isoform of LipL32 was excised from each gel (as determined by alignment of gel images) for analysis by LC-MS/MS.

### In-gel trypsin digestion of IVCL and RUIL LipL32 spots and LC-MS/MS analysis

Excised LipL32 spots were digested with trypsin and analyzed using a QSTAR Pulsar I Hybrid Quadrupole-TOF liquid chromatography-electrospray ionization-tandem mass spectrometer (LC-MS/MS) as previously described [17].

### Data analysis

MS/MS data were searched by centroiding with Analyst QS 1.1 Mascot script 1.6 b24 (MDS Sciex) to create the Mascot generic format file (.mgf) for data base searching. The.mgf files were searched against the *L. interrogans* proteome in UniProtKB annotation (http://www.uniprot.org/uniprot/?query=leptospira+interrogans+serovar+copenhageni&sort=score&format=*) that was maintained in-house at the University of Victoria-Genome BC Proteomics Centre. Search parameters used were as follows: fixed modification was set to carbamidomethyl (C) and variable modifications were set to methyl (D/E/K), tri-methyl (K), acetyl (K), peptide mass tolerance +0.3 Da, fragment mass tolerance +0.15 Da and trypsin enzyme specificity with 2 missed cleavages. Peptides with ion scores at or above the significance threshold (set at 28) were included in the analysis. Post-translational modifications comprising tri-methylations and acetylations were confirmed via manual *de novo* sequencing using the user input sequence for a given peptide spectrum. To be classified as a confirmed PTM and to identify the precise location of the PTM within the peptide, at least one of the following requirements needed to be achieved: (1) the presence of a neutral loss ion (MH^+^-59) unique for a tri-methylated lysine [34], [35]; (2) the presence of spectral ions encompassing the modified residue and the residue immediately preceding the modified residue, with confirmation that the former contained the PTM and the latter lacked the PTM; or (3) the presence of spectral ions derived from the residue immediately preceding the modified residue and the residue immediately following the modified residue, with confirmation that the former lacked the PTM, the latter included the PTM and did not constitute a residue that could be modified by a tri-methyl or acetyl group, and cumulative modifications could not result in a *Δ m/z* of 42 daltons.

### ELISA analyses

Two LipL32 peptides corresponding to previously identified LipL32 immunodominant epitopes [36] that were identified through MS/MS analyses as containing confirmed PTMs (see requirements outlined above) were synthesized by either GenScript (Piscataway, NJ, USA) or ChinaPeptides (Shanghai, China). For each peptide, unmodified and tri-methylated versions were synthesized (Table 1). Ninety six-well MaxiSorp plates (Nalge-Nunc, Rochester, NY, USA) were coated overnight at 37°C in triplicate with 4 µg per well of either unmodified or modified LipL32 peptides or, as a negative control, an unrelated peptide from the *Treponema pallidum* Tp0751 protein sequence [37]. Wells were blocked with 4% skim milk powder/PBS pH 7.0 for 1.5 hours at room temperature and washed four times with PBS pH 7.0 containing 0.05% Tween-20 (PBST). Wells were incubated for 1.5 hours at room temperature with either a 1∶100 dilution of pooled serum prepared in blocking buffer collected from patients with laboratory-confirmed leptospirosis (n = 15) [5] or, as a positive control, a 1∶5000 dilution of polyclonal rabbit anti-LipL32 serum [38] prepared in blocking buffer. After washing 6 times with PBST, wells were incubated for 1.5 hours at room temperature with a 1∶3000 dilution of either goat anti-human IgG (Fab specific)-peroxidase or goat anti-rabbit IgG (whole molecule) F(ab′)_2_ fragment-peroxidase (both purchased from Sigma-Aldrich, Oakville, Ontario, Canada). Wells were washed six times with PBST and developed at room temperature with the TMB peroxidase substrate system (Kirkegaard & Perry Laboratories, Gaithersburg, MD, USA). Plates were read at 600 nm with a Synergy HT plate reader (BioTek, Winooski, VT, USA), and statistical analyses were performed using the Student's two-tailed *t*-test.

**Table 1:** LipL32 synthetic peptides generated for immune recognition studies.

Peptide	Amino Acid Sequence	Modification
P1	AAKAKPVQKLDDDDDGDDTYKEERHNK	none
P1-K^152^Me^3^	AAKAKPVQKLDDDDDGDDTY**K**EERHNK	tri-methylation on K^152^
P2	LTRIKIPNPPKSFDDLKNIDTKKL	none
P2-K^178^Me^3^	LTRIKIPNPPKSFDDL**K**NIDTKKL	tri-methylation on K^178^

### LipL32 structural analysis

Annotation of the identified lysine modifications within the solved Ca^2+^-bound LipL32 structure [39] was accomplished using the PyMOL Molecular Graphics System, Schrödinger, LLC., available at http://www.pymol.org/pymol.

## Results

### Identification of post-translational modifications of lysine residues within LipL32 under *in vivo* growth conditions

**Figure 1:**
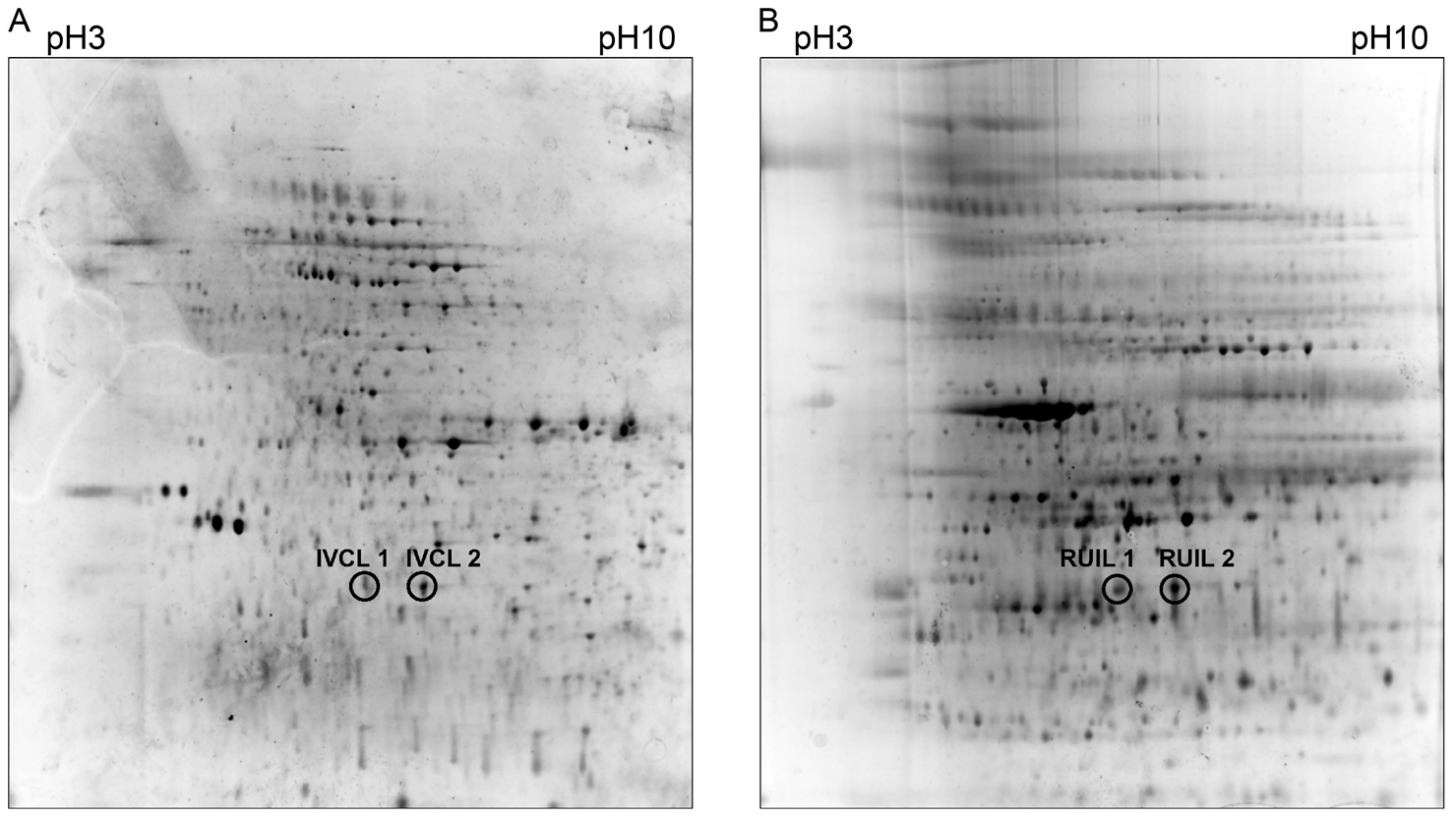
Two-dimensional gel electrophoresis (2DGE) of *Leptospira* whole proteome profiles indicating LipL32 protein spots analyzed by MS/MS. (**A**) 2DGE profile of *in vitro*-cultured *Leptospira* (IVCL) and (**B**) 2DGE profile of rat urine-isolated *Leptospira* (RUIL). Protein derived from whole cells were separated using immobilized pH gradient IPG strips (pH 3–10, non-linear) followed by SDS-PAGE. Circled protein spots corresponding to LipL32 were excised for analysis by LC-MS/MS.

In the current study we investigated the presence of PTMs, with a focus upon methylations, occurring in LipL32 from *L. interrogans* serovar Copenhageni cells grown under *in vivo* and *in vitro* growth conditions. Total protein was prepared from leptospires shed in the urine of rats housed in metabolic cages (rat urine-isolated *Leptospira*; RUIL) and from leptospires cultured in EMJH medium (*in vitro*-cultivated *Leptospira*; IVCL), and each of the whole cell proteomes was independently subjected to two-dimensional gel electrophoresis (Figure 1). Two protein spots corresponding to LipL32 were excised from each of the RUIL and IVCL samples; to allow for a direct comparison, the same LipL32 isoform was selected from each gel. The protein spots were digested with trypsin and analyzed by LC-MS/MS to verify the identity of the excised proteins. The resultant spectrometry data was subjected to manual analysis to confirm detected PTM position assignments. A list of peptides identified by LC-MS/MS, including peptides containing confirmed PTMs, is shown in Table 2, and the obtained MS/MS data and spectra for peptides containing confirmed PTMs can be found in the Supplementary Data Files (Table S1 and Figure S1, respectively).

**Table 2:** Identity and confirmed post-translational modification status of detected LipL32 peptides.

Sample				Peptide sequence*	Modification	Location***
IVCL		RUIL				
1	2	1	2			
-	-	√	√	SSFVLSEDTIPGTNETV**K**	acetylation or tri-methylation	K^29^
-	-	√**	-	LDDDDDGDDTY**K**EER	tri-methylation**	K^152^
-	-	-	√	I**K**IPNPPK	acetylation or tri-methylation	K^166^
-	-	√	-	IPNPP**K**SFDDLK	acetylation or tri-methylation	K^172^
-	-	√	√**	SFDDL**K**NIDTK	acetylation or tri-methylation (RUIL1) or tri-methylation** (RUIL2)	K^178^
-	-	√	√	ISFTTY**K**PGEVK	acetylation or tri-methylation	K^199^
-	-	√	√	QAIAAEESL**K**K	acetylation or tri-methylation	K^245^
-	-	-	√**	QAIAAEESLK**K**	tri-methylation**	K^246^
√	√	√	√	SSFVLSEDTIPGTNETVK	-	-
√	√	√	√	TLLPYGSVINYYGYVKPGQAPDGLVDGNK	-	-
-	√	√	√	TLLPYGSVINYYGYVKPGQAPDGLVDGNKK	-	-
√	-	√	-	LDDDDDGDDTYK	-	-
√	√	√	√	LDDDDDGDDTYKEER	-	-
-	-	√	√	IKIPNPPK	-	-
-	√	-	√	SFDDLKNIDTK	-	-
√	√	√	√	ISFTTYKPGEVK	-	-
√	√	√	√	GSFVASVGLLFPPGIPGVSPLIHSNPEELQK	-	-
√	√	√	√	QAIAAEESLK	-	-
√	√	√	√	QAIAAEESLKK	-	-

Peptides detected within *in vitro*-cultured *Leptospira* (IVCL) and rat urine-isolated *Leptospira* (RUIL) 2DGE samples are listed.

*modified lysine residues are indicated by underlined bold font.

**confirmed tri-methylation indicated by the presence of the M^+^-59 neutral loss ion.

***refers to the amino acid residue containing the modified lysine residue. The identified amino acid residue corresponds to the location within the mature LipL32 protein sequence.

High protein coverage was obtained for the two LipL32 protein spots excised from each of the IVCL and RUIL samples (designated as IVCL1, IVCL2, RUIL1 and RUIL2; see Table 2). Combined, the detected peptides covered 45% (IVCL1 and RUIL1) and 47% (IVCL2 and RUIL2) of the LipL32 sequence. Analysis of PTMs observed within the RUIL samples identified eight peptides containing modifications, whereas no modifications were detected in the IVCL samples. All modifications were confirmed by manual analysis of the mass spectra and were exclusively observed on lysine residues. The observation that five out of eight of the modified lysine residues were detected in peptides containing a missed trypsin cleavage (Table S1) is noteworthy, for the presence of lysine modifications is known to reduce the cleavage efficiency of trypsin by masking the enzyme substrate site [34]. Thus, the number of lysine modifications detected within LipL32 in this study may be under-representative of the actual number present within this protein.

For five of the eight modified peptides that were detected, the nature of the PTMs could only be narrowed down to either tri-methylation (*Δ m/z* 42.0471) or acetylation (*Δ m/z* 42.0106). Due to the highly similar *m/z* ratios obtained for tri-methylation and acetylation, differentiation of these two modifications via mass spectrometry relies upon detection of the neutral loss ion (MH^+^-59) which is unique for a tri-methylated lysine [34], [35]. In our analyses, three peptides contained MH^+^-59 neutral loss ions (LDDDDDGDDTYKEER from RUIL1, SFDDLKNIDTK from RUIL2, and QAIAAEESLKK from RUIL2; Table 2). For each peptide the presence of the neutral loss ion on a fragment containing the modified lysine, combined with the presence of spectral ions surrounding the modified lysine, enabled precise residue assignment of the tri-methyl group addition to residues K^152^, K^178^ and K^246^ (Figure S1). Of note, a modified version of the LDDDDDGDDTYKEER peptide was also detected in the RUIL2 sample. Although the spectra obtained for this peptide prevented assignment of a precise location for the modification, it could be narrowed down to the region of the peptide encompassing the sequence TYKE (residues 150–153; Figure S1). For seven out of eight of the modified peptides detected within RUIL1 and RUIL2 the corresponding unmodified peptide was also detected, indicating that the observed modifications were not ubiquitous within the LipL32 spots selected for analysis. No further PTMs were detected within either the IVCL or RUIL samples.

### Leptospirosis patient serum reactivity against modified and unmodified versions of synthetic LipL32 peptides

**Figure 2:**
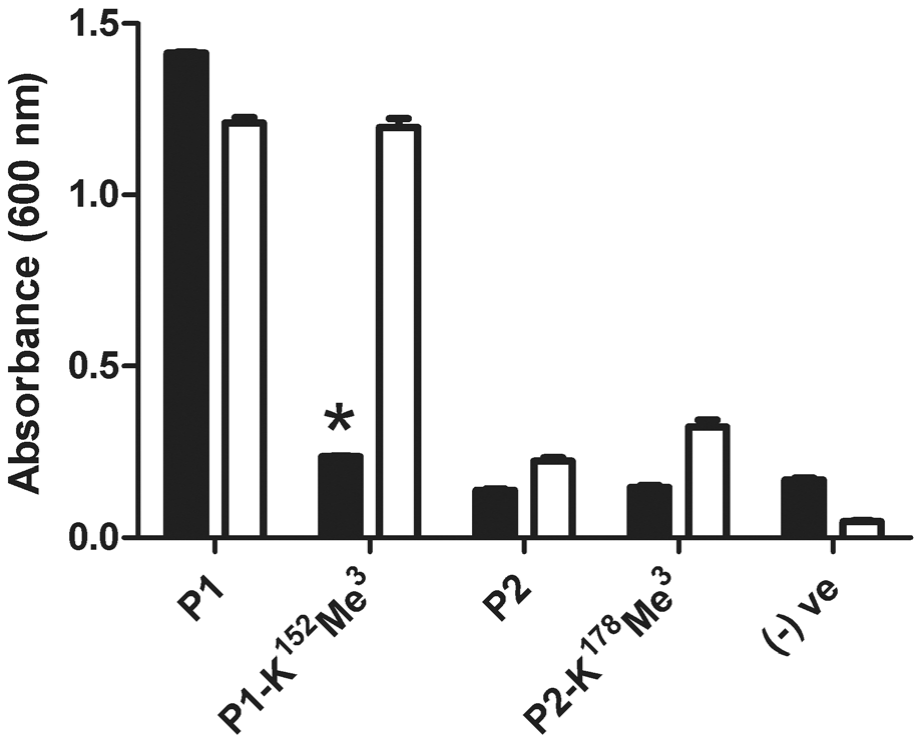
Leptospirosis patient serum reactivity against modified and unmodified versions of synthetic LipL32 peptides. Shown is the reactivity of pooled sera from laboratory-confirmed leptospirosis patients (black bars) and LipL32-specific polyclonal rabbit serum (white bars) to unmodified and tri-methylated (KMe^3^) versions of P1 and P2 peptides comprising experimentally-determined B-cell epitopes of LipL32 [36]. Two independent experiments were performed with reproducible results; the results from one representative experiment are shown. Error bars represent standard error of measurement from triplicate samples. For statistical analyses, the level of reactivity of patient sera against the unmodified version of P1 was compared to the level of reactivity against the P1 peptide containing a tri-methylation at position K^152^ (P1-K^152^Me^3^) using the Student's two-tailed *t*-test (*p<0.0001).

Analysis of the modified LipL32 peptides detected within the RUIL samples highlighted four peptides that overlap with regions previously identified through epitope mapping studies as antigenic regions of LipL32 [36]. Within the reported LipL32 epitope spanning residues 132–158 of the mature protein (designated as P1; AAKAKPVQKLDDDDDGDDTYKEERHNK) we detected tri-methylation of residue K^152^. For the LipL32 epitope encompassing residues 162–185 of the mature protein (designated as P2; LTRIKIPNPPKSFDDLKNIDTKKL) we detected modifications on K^166^, K^172^ and K^178^, with the latter constituting a confirmed tri-methylation. In order to investigate if the modifications altered immune recognition of these epitopes, synthetic peptides corresponding to the two previously identified epitopes were prepared; specifically, unmodified and K^152^ tri-methylated versions of P1 were synthesized, while for P2 an unmodified and a representative modified peptide containing a tri-methylated K^178^ residue were synthesized (Table 1). When tested in an ELISA assay (Figure 2), pooled sera obtained from patients with laboratory-confirmed leptospirosis reacted strongly to the unmodified version of P1, while reactivity against the K^152^ tri-methylated peptide version was significantly decreased (*p*<0.0001). As a control, polyclonal antiserum raised against recombinant LipL32 was tested for reactivity against the unmodified and modified versions of P1; interestingly, no difference in reactivity was observed.

In our study minimal reactivity of both patient sera and rabbit serum was observed against the unmodified and modified versions of P2, a result that differs from the reactivity observed by Lottersberger *et al.* against P2 using hyperimmune rabbit serum [36]. Assessment of the coated P1/P2 peptide concentrations demonstrated no difference in coating efficiency between the peptides, indicating this result was not due to inadequate coating of P2 within the ELISA wells. Due to the minimal reactivity observed against the unmodified version of P2, the effect of modification of P2 on immune recognition of this epitope could not be assessed.

### Location of PTMs within the Ca^2+^-bound LipL32 structure

**Figure 3:**
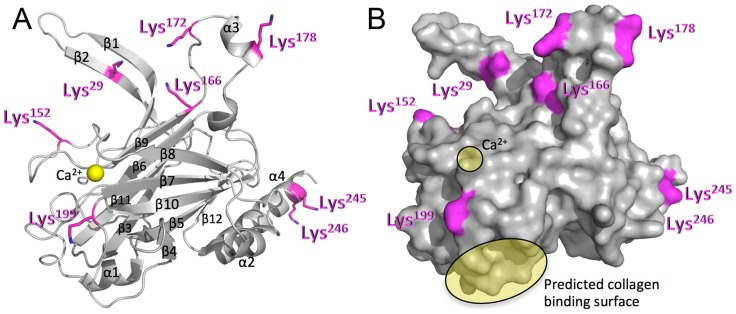
Modified lysine residues mapped onto the Ca^2+^ bound LipL32 structure [39]. **A.** Secondary structure and **B.** surface representations. The numbering system C^1^-K^253^ corresponds to the mature LipL32 protein (lacking the 19 residue cleaved SPII signal sequence) established by Vivian *et al.* [40] and adopted by Tung *et al.* [39]. Note, the Ca^2+^ binding site and predicted collagen binding surface in panel **B** are highlighted in yellow and frame Lys199. The figure was generated using PyMOL (Molecular Graphics System, Version 1.5.0.4 Schrödinger, LLC).

The structural position of the eight modified lysines detected within the LipL32 sample originating from the rat urine-isolated leptospires was determined through examination of the Ca^2+^-bound LipL32 crystal structure [39]. For consistency and ease of interpretation, we have used the numbering system C^1^-K^253^ which corresponds to the mature LipL32 protein (lacking the 19 residue cleaved SPII signal sequence) set forward by Vivian *et al.* [40] and adopted by Tung *et al.* [39]. As shown in Figure 3A, the observed modifications were localized to the β2 strand (K^29^), the loop between the β8 and β9 strands (K^152^), the β9 strand (K^166^), the loop between the β9 strand and the α3 helix (K^172^), the α3 helix (K^178^), the loop between the β10 and β11 strands (K^199^) and the α4 helix (K^245^ and K^246^). All eight modified lysines localized to surface accessible regions of the structure (Figure 3B). Of note, the modified residues K^152^ and K^199^ are located in immediate proximity to the LipL32 Ca^2+^-binding site, which comprises the negatively charged surface formed by residues D^142^–D^149^ (encompassing a portion of the β8 strand and the β8β9 loop) and the Ca^2+^ ion-coordinating residues in the α1β7 loop (D^113^ and T^114^) and the β8β9 loop (D^145^, D^146^ and Y^159^) [39]. Lysine 199 is also in close proximity to the reported collagen-binding site encompassing residues L^53^, V^54^, Y^62^, W^115^, R^117^, Y^151^ and Y^198^ [40] (Figure 3B). In fact, K^199^ is positioned on a flexible loop between strands 10 and 11 that bridges the Ca^2+^ and reported collagen-binding sites of LipL32, suggesting that modification of K^199^ may play a role in regulating protein architecture and function.

## Discussion

Two previous proteomic studies have established the presence of post-translationally modified proteins within *L. interrogans*. The leptospiral protein OmpL32 (corresponding to LIC11848) was shown to be differentially methylated on glutamic acid residues from *L. interrogans* grown under *in vitro* conditions [17]. Additionally, a global PTM analysis of *in vitro*-cultured *L. interrogans* serovar Lai detected multiple PTMs on a broad range of proteins, including 46 proteins with 54 lysine acetylation sites, 104 proteins with 135 glutamic acid/glutamine methylation sites, and 58 proteins with 64 lysine/arginine methylation sites. One of the lysine-modified proteins detected within the latter study was LipL32, which was shown to be devoid of lysine acetylations but to contain mono-, di-, and tri-methyl groups on residue K^152^ [18]. In the study presented herein, we similarly detected modification of residue K^152^, as well as seven other lysine residues, within LipL32 from *in vivo*-grown leptospires. Of the modified lysines that were detected an unambiguous assignment of tri-methylation could be made for only residues K^152^, K^178^ and K^246^. By extrapolation from these results it is plausible that the remaining five detected PTMs constitute similar lysine tri-methylations, however definitive assignment could not be made from the spectra obtained in this study.

The prior proteomic studies outlined above establish that post-translational protein modification is a phenomenon which occurs within *in vitro*-cultured *L. interrogans*. Our study extends this observation and establishes that, at least for modification of lysine residues within the major leptospiral protein LipL32, this phenomenon also occurs under biologically relevant *in vivo* infection conditions. Of particular interest is the fact that in our study modification of LipL32 lysine residues was restricted to *in vivo*-grown organisms, while in the study performed by Cao and colleagues LipL32 lysine modification was observed at K^152^ within *in vitro*-cultured *Leptospira* [18]. These divergent results may stem from the use of different strains in the two *in vitro* studies (Lai in the Cao *et al.* study, Copenhageni in our study). Alternatively, the difference may have arisen due to the higher *in vitro* culture density attained for *L. interrogans* in the Cao *et al.* study, which reached approximately 6.6×10^8^ bacteria/ml compared to 1×10^8^ bacteria/ml in our study. The higher density may be more representative of that achieved during kidney colonization by *Leptospira* and thus may indicate the process of protein modification is dependent, at least partially, upon elevated bacterial density. Such a phenomenon has recently been described for *Pseudomonas aeruginosa* with respect to the PrmC methyltransferase, a protein that mediates methylation of key virulence factors and whose activity is controlled by quorum sensing [41].

In this study we observed a correlation between lysine modifications occurring within LipL32 and regions of the protein that have been previously identified as immunogenic. Seven out of eight of the identified lysine modifications occurred in regions identified by Hauk and colleagues as being reactive with patient sera [42]. Of particular interest, six of the modifications occurred in the region of the mature protein encompassing amino acids 166–253 that was shown by Hauk *et al.* to be most strongly reactive with acute phase sera [42]. Further, four out of eight of the identified lysine modifications occurred in regions previously identified as epitopes via peptide mapping studies [36]. Our subsequent demonstration that modification of K^152^ within one of these epitopes significantly decreased the reactivity of leptospirosis patient serum compared to the unmodified epitope version suggests the possibility that lysine tri-methylation within LipL32 may decrease immune recognition of this protein during infection. Supporting this theory is our finding that decreased reactivity was solely observed using patient sera and not rabbit serum raised against recombinant LipL32, suggesting the decreased immune recognition of the modified LipL32 epitope is a phenomenon observed only under conditions of infection. Additionally, the modifications present within *Leptospira* isolated from rats may differ from those arising during infection in humans, a possibility that would further explain the decreased immune recognition of patient sera against a rat-derived modified LipL32 peptide.

An alternative, or additional, proposed function for LipL32 lysine modification comes from determination of the location of the modified residues within the solved LipL32 structure. LipL32 has been shown to possess a calcium-binding cluster that comprises residues D^113^, T^114^, D^145^, D^146^, and Y^159^, with the latter three residues being localized to the β8β9 loop within the LipL32 structure [39]. The β8β9 loop undergoes a significant conformational change upon calcium binding, a shift that has been suggested to account for the enhanced stability observed in the LipL32 calcium-bound state [39], [43]. The modification observed within our study at K^152^ similarly lies within the β8β9 loop. The relatively large size and charge modification of a tri-methyl group at K^152^ and either an acetyl or tri-methyl modification at K^199^, combined with the close proximity to the Ca^2+^-coordinating residues D^145^, D^146^ and Y^159^, has the potential to affect Ca^2+^ binding. This may affect the stability of LipL32 and/or impact LipL32 functions that are linked to calcium binding, such as the putative immune system modulatory function that has been ascribed to the calcium-bound state of LipL32 (promotion of interaction with the Toll-like receptor 2 to induce an inflammatory response) [44]. Whether methylation could influence binding of LipL32 to extracellular matrix components, which has been shown by Hauk *et al.* to be independent of calcium binding [45], or other currently unknown LipL32 functions remains to be seen.

Although originally identified as a surface-exposed outer membrane lipoprotein, a recent study using multiple, independent methods has suggested that LipL32 has limited surface exposure [46]. If discrete sub-surface and surface-exposed LipL32 subpopulations exist, PTMs incorporated into the latter may contribute to immune evasion, as hypothesized in the current study. Interestingly, we detected both unmodified and modified versions of the identified LipL32 peptides from *in vivo*-isolated organisms, indicating that subpopulations of modified/unmodified LipL32 proteins are present during the course of an infection. These could correspond to distinct subpopulations comprised of surface-exposed/modified and sub-surface/unmodified LipL32 molecules. Additionally, since the RUIL samples analyzed in this study constitute a pool collected over a six week time frame, PTMs could have arisen over time in response to immune pressure, with a higher proportion of modified LipL32 being present at later time points. Both scenarios suggest LipL32 modification could play a role in establishment of persistent infection. However, a study by Murray and co-workers showed no reduction in the ability of an *L. interrogans lipL32* transposon mutant to colonize rats [47]. Although this result is seemingly at odds with our proposed link between LipL32 modification, immune evasion and leptospiral persistence, the ability of the *lipL32* mutant to persist beyond 15 days post-infection was not assessed, thus leaving open the possibility that wild-type and LipL32-deficient *Leptospira* may exhibit differences in the capacity to establish long-term infection within rats.

The discovery of LipL32 lysine modification in organisms shed in the urine has potential implications for understanding how leptospires persist for extended periods of time in the renal tubules. Such persistence is responsible for the ability of leptospires to maintain high rates of shedding, transmission, and infection among reservoir host populations. An unanswered paradox in leptospiral biology is why leptospires exhibit high levels of LipL32 expression in the renal tubules [38] despite a robust LipL32 immune response during infection [48], [49]. The data presented here provide a partial explanation for this paradox by showing that serum from infected patients recognized a methylated LipL32 peptide far less well than the unmethylated form. This result indicates that lysine methylation sufficiently alters peptide epitopes as to prevent antibody binding. Since PTMs have also been shown to alter immunologic processing and presentation of peptides in the context of MHC class I [50], [51], the cell-mediated arm of the immune system may similarly exhibit an altered response to modified compared to unmodified LipL32. Of interest in this regard, an IFN-γ-stimulating T cell epitope has recently been detected within LipL32 and has been shown to reside between amino acids 1 and 181 [52], a region that corresponds to five of the eight modified lysines detected in this study (K^29^, K^152^, K^166^, K^172^, K^178^). The possibility exists that LipL32 modification decreases both humoral and cell-mediated immune recognition of the protein, thus preventing effective bacterial clearance and promoting leptospiral persistence. The importance of prevention of renal tubule colonization with respect to protection from leptospiral infection is exemplified by the study performed by Seixas *et al.* In this study, hamsters immunized with *Mycobacterium bovis* bacillus Calmette-Guérin (BCG)-expressing LipL32 were more likely to survive *Leptospira* challenge than hamsters immunized with BCG alone, and enhanced survival corresponded with an absence of renal tubule colonization [53]. Collectively, these studies suggest that an increased understanding of how PTMs affect the humoral and cell-mediated immune responses to LipL32 and, relatedly, whether modification of LipL32 contributes to bacterial persistence, may be key to development of vaccines that are able to generate sterilizing immunity.

In summary, this study reports the detection of multiple modified lysine residues within the major leptospiral protein LipL32 isolated from leptospires grown under *in vivo*, but not *in vitro*, conditions and provides insight into the potential functional and immunological consequences of these modifications. In addition this study provides a potential molecular mechanism for, and link between, establishment of chronic *Leptospira* infection in rats and acute *Leptospira* infection in humans. Of importance to assess through future studies are the temporal occurrence, prevalence, and biological relevance of LipL32 lysine modifications at both the cellular and community levels, as well as whether PTMs are detected on other leptospiral OMPs during the course of infection. These investigations may uncover the elusive function of this highly abundant leptospiral protein and may reveal a novel mechanism of bacterial immune evasion.

## Supporting Information

Figure S1 **MS/MS spectra for peptides containing a modified lysine.** Modified lysines are underlined in the peptide sequence. MH^+^-59 ions, which are neutral loss ions indicative of peptides containing a tri-methylated lysine [35], are indicated in blue boxes within the spectra.
(PDF)

Table S1 **MS/MS data for unmodified and modified peptides detected in IVCL and RUIL LipL32 protein spots.** Peptides containing a confirmed modified lysine residue are shaded grey, and the modified residue is bolded and underlined in the peptide sequence.
(XLSX)
