# Supplementary material for: Post-translational Modification of LipL32 during Leptospira interrogans Infection
Source: PLoS Negl Trop Dis. 2014 Oct 30;8(10):e3280. doi: 10.1371/journal.pntd.0003280 (PMC4214626; doi:10.1371/journal.pntd.0003280)

RUIL - Spot 1 - LipL32

Sequence: **QAIAAEESLKK**
MH+: 1228.716788
Charge: 2+
Ions Score: 79
Expect: 7.5e-007

#	b	b++	b*	b*++	b0	b0++	Seq.	y	y++	y*	y*++	y0	y0++	#
**1**	129.0659	65.0366	112.0393	56.5233			**Q**							**11**
**2**	**200.1030**	100.5551	183.0764	92.0418			**A**	***1101.6150***	551.3111	1084.5885	542.7979	1083.6045	542.3059	**10**
**3**	**313.1870**	157.0972	296.1605	148.5839			**I**	***1030.5779***	515.7926	1013.5514	507.2793	1012.5673	506.7873	**9**
**4**	**384.2241**	192.6157	367.1976	184.1024			**A**	***917.4938***	459.2506	900.4673	450.7373	899.4833	450.2453	**8**
**5**	**455.2613**	228.1343	438.2347	219.6210			**A**	***846.4567***	423.7320	829.4302	415.2187	828.4462	414.7267	**7**
**6**	584.3039	292.6556	567.2773	284.1423	566.2933	283.6503	**E**	***775.4196***	388.2134	758.3931	379.7002	757.4090	379.2082	**6**
**7**	**713.3464**	357.1769	696.3199	348.6636	695.3359	348.1716	**E**	***646.3770***	323.6921	629.3505	315.1789	628.3665	314.6869	**5**
**8**	800.3785	400.6929	783.3519	392.1796	782.3679	391.6876	**S**	***517.3344***	259.1709	500.3079	250.6576	499.3239	250.1656	**4**
**9**	913.4625	457.2349	896.4360	448.7216	895.4520	448.2296	**L**	***430.3024***	215.6548	413.2758	207.1416			**3**
**10**	**1083.5681**	542.2877	1066.5415	533.7744	1065.5575	533.2824	**K**	***317.2183***	159.1128	300.1918	150.5995			**2**
**11**							**K**	***147.1128***	74.0600	130.0863	65.5468			**1**

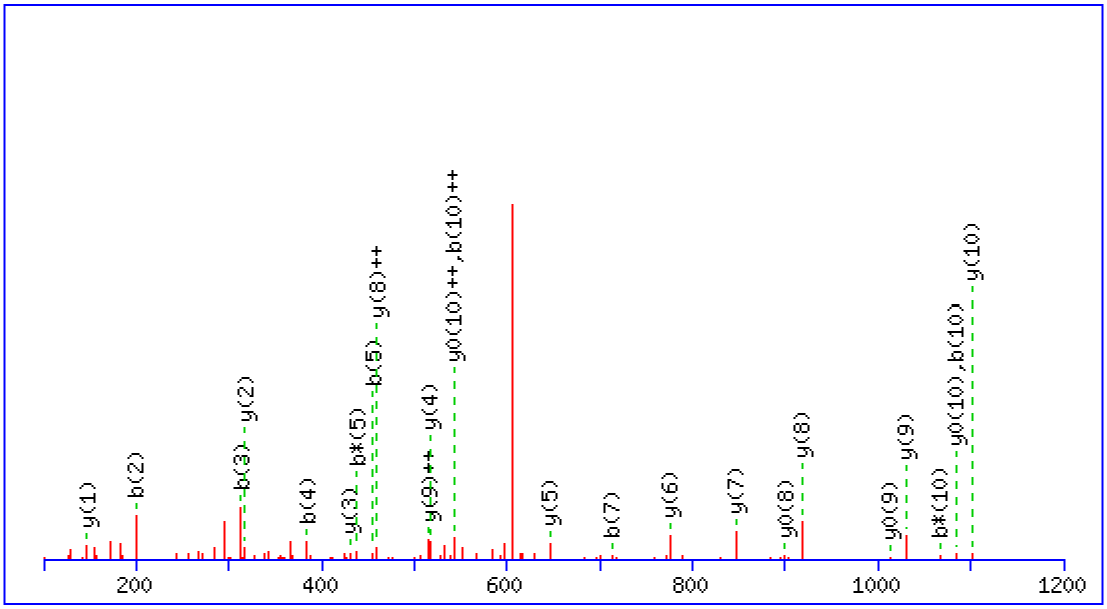

RUIL - Spot 1 - LipL32

Sequence: **SFDDLKNIDTK**
MH+: 1336.696720
Charge: 2+
Ions Score: 58
Expect: 6.2e-005

#	b	b++	b*	b*++	b0	b0++	Seq.	y	y++	y*	y*++	y0	y0++	#
**1**	88.0393	44.5233	70.0287	35.5180			**S**							**11**
**2**	235.1077	118.0575	217.0972	109.0522			**F**	1250.6263	625.8168	1233.5998	617.3035	1232.6157	616.8115	**10**
**3**	***350.1347***	175.5710	332.1241	166.5657			**D**	***1103.5579***	552.2826	1086.5313	543.7693	1085.5473	543.2773	**9**
**4**	***465.1616***	233.0844	447.1510	224.0792			**D**	***988.5310***	494.7691	971.5044	486.2558	970.5204	485.7638	**8**
**5**	578.2457	289.6265	560.2351	280.6212			**L**	***873.5040***	437.2556	856.4775	428.7424	855.4934	428.2504	**7**
**6**	***748.3512***	374.6792	731.3246	366.1660	730.3406	365.6740	**K**	***760.4199***	380.7136	743.3934	372.2003	742.4094	371.7083	**6**
**7**	***862.3941***	431.7007	845.3676	423.1874	844.3836	422.6954	**N**	***590.3144***	295.6608	573.2879	287.1476	572.3039	286.6556	**5**
**8**	***975.4782***	488.2427	958.4516	479.7295	957.4676	479.2374	**I**	476.2715	238.6394	459.2449	230.1261	458.2609	229.6341	**4**
**9**	***1090.5051***	545.7562	1073.4786	537.2429	1072.4946	536.7509	**D**	***363.1874***	182.0974	346.1609	173.5841	345.1769	173.0921	**3**
**10**	***1191.5528***	596.2800	1174.5263	587.7668	1173.5422	587.2748	**T**	***248.1605***	124.5839	231.1339	116.0706	230.1499	115.5786	**2**
**11**							**K**	***147.1128***	74.0600	130.0863	65.5468			**1**

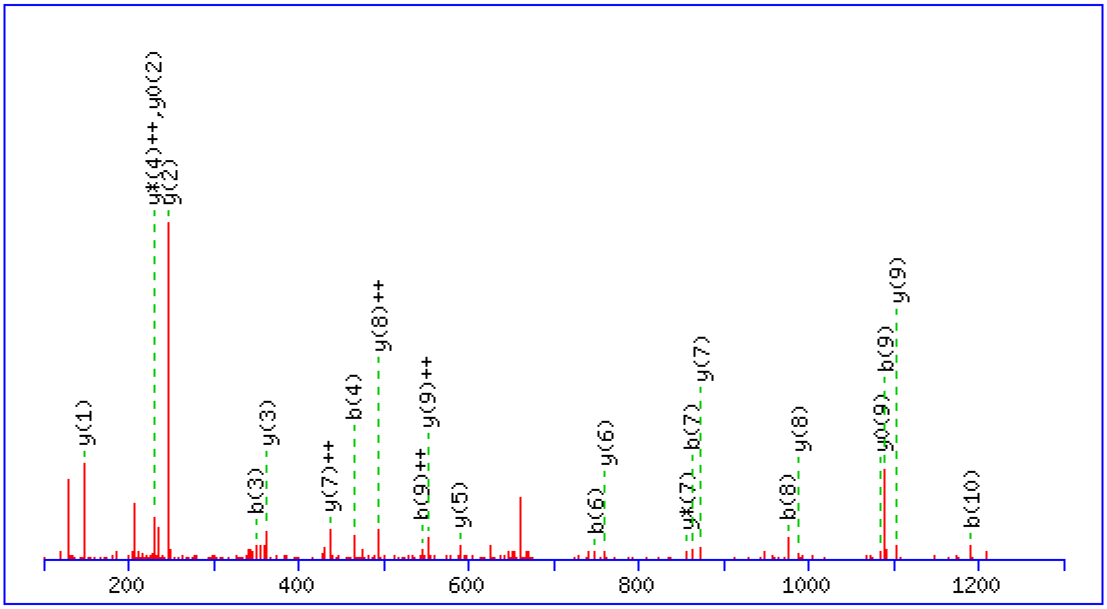

RUIL - Spot 1 - LipL32

Sequence: **ISFTTYKPGEVK**
MH+: 1410.793130
Charge: 2+
Ions Score: 61
Expect: 4.3e-005

#	b	b++	b*	b*++	b0	b0++	Seq.	y	y++	y*	y*++	y0	y0++	#
**1**	114.0913	57.5493					**I**							**12**
**2**	201.1234	101.0653	183.1128	92.0600			**S**	1298.6627	649.8350	1281.6361	641.3217	1280.6521	640.8297	**11**
**3**	***348.1918***	174.5995	330.1812	165.5942			**F**	***1211.6307***	606.3190	1194.6041	597.8057	1193.6201	597.3137	**10**
**4**	***449.2395***	225.1234	431.2289	216.1181			**T**	***1064.5623***	532.7848	1047.5357	524.2715	1046.5517	523.7795	**9**
**5**	550.2871	275.6472	532.2766	266.6419			**T**	***963.5146***	482.2609	946.4880	473.7477	945.5040	473.2556	**8**
**6**	713.3505	357.1789	695.3399	348.1736			**Y**	***862.4669***	431.7371	845.4403	423.2238	844.4563	422.7318	**7**
**7**	***883.4560***	442.2316	866.4294	433.7184	865.4454	433.2264	**K**	699.4036	350.2054	682.3770	341.6921	681.3930	341.2001	**6**
**8**	980.5088	490.7580	963.4822	482.2447	962.4982	481.7527	**P**	***529.2980***	265.1527	512.2715	256.6394	511.2875	256.1474	**5**
**9**	***1037.5302***	519.2688	1020.5037	510.7555	1019.5197	510.2635	**G**	432.2453	216.6263	415.2187	208.1130	414.2347	207.6210	**4**
**10**	***1166.5728***	583.7900	1149.5463	575.2768	1148.5623	574.7848	**E**	375.2238	188.1155	358.1973	179.6023	357.2132	179.1103	**3**
**11**	***1265.6412***	633.3243	1248.6147	624.8110	1247.6307	624.3190	**V**	***246.1812***	123.5942	229.1547	115.0810			**2**
**12**							**K**	***147.1128***	74.0600	130.0863	65.5468			**1**

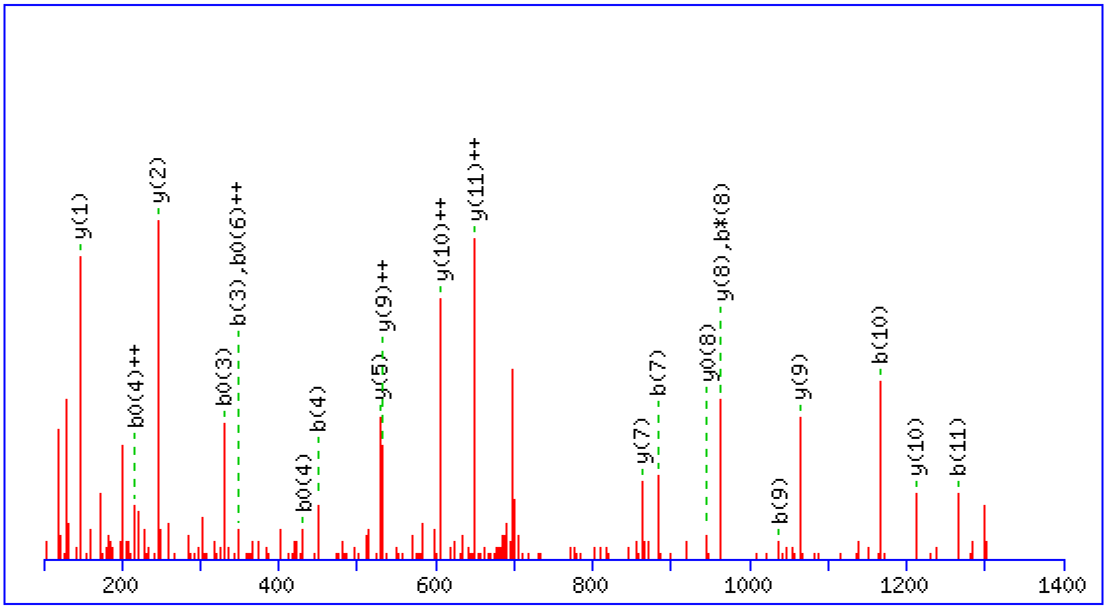

RUIL - Spot 1 - LipL32

Sequence: **IPNPPKSFDDLK**
MH+: 1411.778895
Charge: 3+
Ions Score: 28
Expect: 0.036

#	b	b++	b*	b*++	b0	b0++	Seq.	y	y++	y*	y*++	y0	y0++	#
1	114.0913	57.5493					I							12
2	**211.1441**	106.0757					P	1299.6579	650.3326	1282.6314	641.8193	1281.6474	641.3273	11
3	**325.1870**	163.0972	308.1605	154.5839			N	1202.6052	601.8062	1185.5786	593.2930	1184.5946	592.8009	10
4	422.2398	211.6235	405.2132	203.1103			P	1088.5623	544.7848	1071.5357	536.2715	1070.5517	535.7795	9
5	519.2926	***260.1499***	502.2660	251.6366			P	***991.5095***	496.2584	974.4829	487.7451	973.4989	487.2531	8
6	689.3981	345.2027	672.3715	336.6894			K	894.4567	447.7320	877.4302	439.2187	876.4462	438.7267	7
7	776.4301	388.7187	759.4036	380.2054	758.4196	379.7134	S	***724.3512***	362.6792	707.3246	354.1660	706.3406	353.6740	6
8	923.4985	462.2529	906.4720	453.7396	905.4880	453.2476	F	637.3192	319.1632	620.2926	310.6499	619.3086	310.1579	5
9	1038.5255	519.7664	1021.4989	511.2531	1020.5149	510.7611	D	490.2508	245.6290	473.2242	237.1157	472.2402	236.6237	4
10	1153.5524	***577.2798***	1136.5259	568.7666	1135.5419	568.2746	D	***375.2238***	188.1155	358.1973	179.6023	357.2132	179.1103	3
11	1266.6365	633.8219	1249.6099	625.3086	1248.6259	624.8166	L	***260.1969***	130.6021	243.1703	122.0888			2
12							K	***147.1128***	74.0600	130.0863	65.5468			1

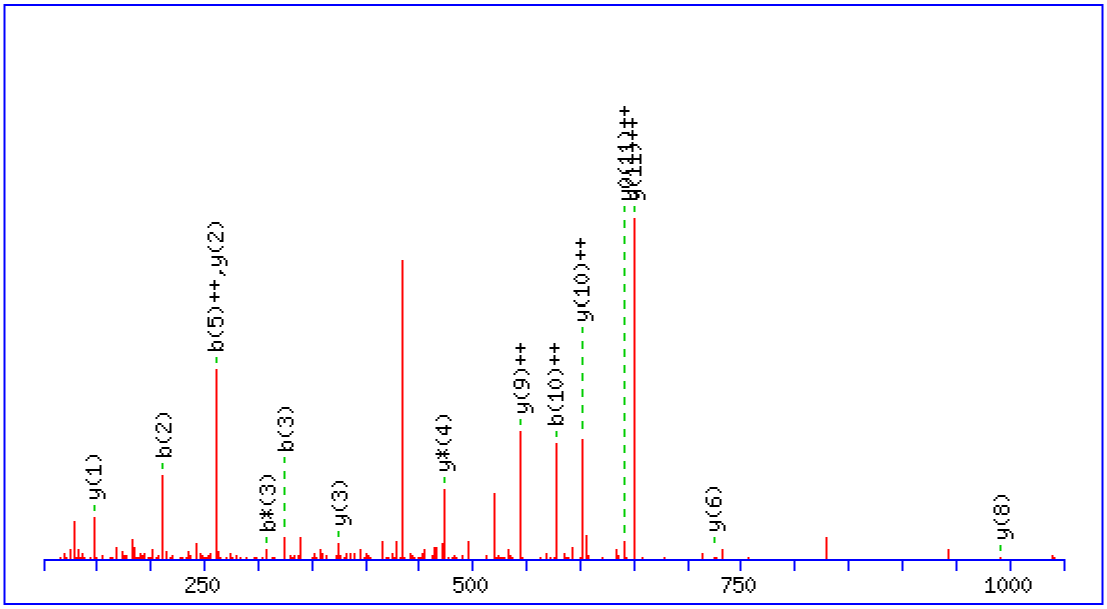

RUIL - Spot 1 - LipL32

Sequence: **LDDDDDGDDTY<u>K</u>EER**
MH+: 1841.778453
Charge: 3+
Ions Score: 32
Expect: 0.0032

#	b	b++	b*	b*++	b0	b0++	Seq.	y	y++	y*	y*++	y0	y0++	#
1	114.0913	57.5493					L							15
2	**229.1183**	115.0628	211.1077	106.0575			D	1729.6671	865.3372	1712.6406	856.8239	1711.6566	856.3319	14
3	**344.1452**	172.5763	326.1347	163.5710			D	1614.6402	807.8237	1597.6136	799.3105	1596.6296	798.8184	13
4	**459.1722**	230.0897	441.1616	221.0844			D	1499.6132	750.3103	1482.5867	741.7970	1481.6027	741.3050	12
5	574.1991	287.6032	556.1885	278.5979			D	1384.5863	692.7968	1367.5597	684.2835	1366.5757	683.7915	11
6	689.2261	345.1167	671.2155	336.1114			D	1269.5594	635.2833	1252.5328	626.7700	1251.5488	626.2780	10
7	746.2475	373.6274	728.2370	364.6221			G	1154.5324	577.7698	1137.5059	569.2566	1136.5218	568.7646	9
8	861.2745	431.1409	843.2639	422.1356			D	1097.5109	549.2591	1080.4844	540.7458	1079.5004	540.2538	8
9	976.3014	488.6543	958.2908	479.6491			D	982.4840	491.7456	965.4575	483.2324	964.4734	482.7404	7
10	1077.3491	539.1782	1059.3385	530.1729			T	867.4571	434.2322	850.4305	425.7189	849.4465	425.2269	6
11	1240.4124	620.7098	1222.4018	611.7046			Y	766.4094	383.7083	749.3828	375.1951	748.3988	374.7030	5
12	1410.5543	705.7808	1393.5278	697.2675	1392.5438	696.7755	K	603.3461	302.1767	586.3195	293.6634	585.3355	293.1714	4
13	1539.5969	770.3021	1522.5704	761.7888	1521.5864	761.2968	E	433.2041	217.1057	416.1776	208.5924	415.1936	208.1004	3
14	1668.6395	834.8234	1651.6130	826.3101	1650.6289	825.8181	E	304.1615	152.5844	287.1350	144.0711	286.1510	143.5791	2
15							R	175.1190	88.0631	158.0924	79.5498			1

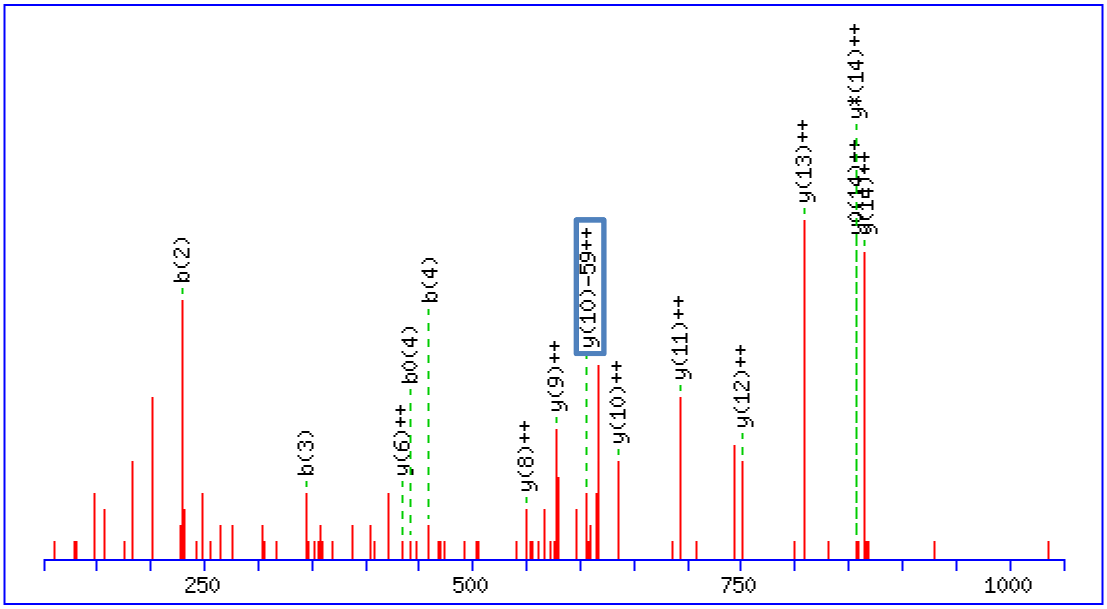

RUIL - Spot 1 - LipL32

Sequence: **SSFVLSEDTIPGTNETVK**
MH+: 1965.027392
Charge: 2+
Ions Score: 84
Expect: 3.8e-008

#	b	b++	b*	b*++	b0	b0++	Seq.	y	y++	y*	y*++	y0	y0++	#
1	88.0393	44.5233	70.0287	35.5180			S							18
2	175.0713	88.0393	157.0608	79.0340			S	1878.9331	939.9702	1861.9066	931.4569	1860.9226	930.9649	17
3	**322.1397**	161.5735	304.1292	152.5682			F	1791.9011	896.4542	1774.8745	887.9409	1773.8905	887.4489	16
4	**421.2082**	211.1077	403.1976	202.1024			V	1644.8327	822.9200	1627.8061	814.4067	1626.8221	813.9147	15
5	**534.2922**	267.6498	516.2817	258.6445			L	1545.7643	773.3858	1528.7377	764.8725	1527.7537	764.3805	14
6	**621.3243**	311.1658	603.3137	302.1605			S	***1432.6802***	716.8437	1415.6536	708.3305	1414.6696	707.8385	13
7	**750.3668**	375.6871	732.3563	366.6818			E	***1345.6482***	673.3277	1328.6216	664.8144	1327.6376	664.3224	12
8	865.3938	433.2005	847.3832	424.1952			D	***1216.6056***	608.8064	1199.5790	600.2932	1198.5950	599.8011	11
9	**966.4415**	483.7244	948.4309	474.7191			T	***1101.5786***	551.2930	1084.5521	542.7797	1083.5681	542.2877	10
10	**1079.5255**	540.2664	1061.5150	531.2611			I	***1000.5310***	500.7691	983.5044	492.2558	982.5204	491.7638	9
11	1176.5783	588.7928	1158.5677	579.7875			P	***887.4469***	444.2271	870.4203	435.7138	869.4363	435.2218	8
12	1233.5998	617.3035	1215.5892	608.2982			G	***790.3941***	395.7007	773.3676	387.1874	772.3836	386.6954	7
13	1334.6474	667.8274	1316.6369	658.8221			T	733.3727	367.1900	716.3461	358.6767	715.3621	358.1847	6
14	1448.6904	724.8488	1431.6638	716.3355	1430.6798	715.8435	N	***632.3250***	316.6661	615.2984	308.1529	614.3144	307.6608	5
15	1577.7330	789.3701	1560.7064	780.8568	1559.7224	780.3648	E	518.2821	259.6447	501.2555	251.1314	500.2715	250.6394	4
16	1678.7806	839.8940	1661.7541	831.3807	1660.7701	830.8887	T	***389.2395***	195.1234	372.2129	186.6101	371.2289	186.1181	3
17	1777.8491	889.4282	1760.8225	880.9149	1759.8385	880.4229	V	***288.1918***	144.5995	271.1652	136.0863			2
18							K	***189.1234***	95.0653	172.0968	86.5520			1

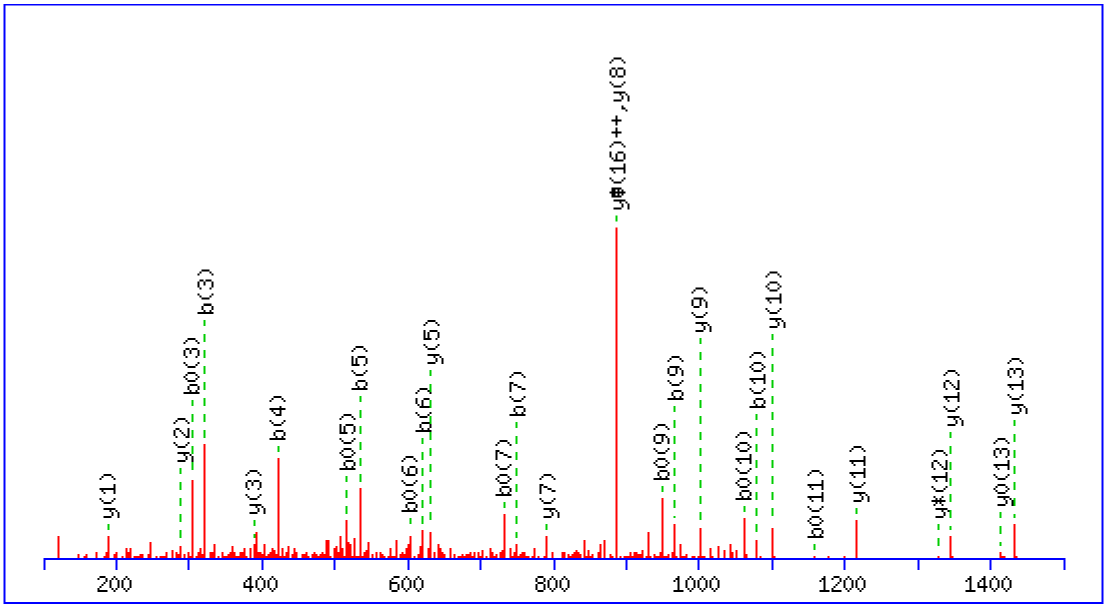

RUIL - Spot 2 - LipL32

Sequence: **IKIPNPPK**
MH+: 947.590206
Charge: 2+
Ions Score: 32
Expect: 0.0033

#	b	b++	b*	b*++	Seq.	y	y++	y*	y*++	#
1	114.0913	57.5493			I					8
2	284.1969	142.6021	267.1703	134.0888	K	***835.5036***	418.2554	818.4771	409.7422	7
3	397.2809	199.1441	380.2544	190.6308	I	***665.3981***	333.2027	648.3715	324.6894	6
4	494.3337	247.6705	477.3071	239.1572	P	***552.3140***	276.6606	535.2875	268.1474	5
5	**608.3766**	304.6919	591.3501	296.1787	N	455.2613	228.1343	438.2347	219.6210	4
6	**705.4294**	353.2183	688.4028	344.7051	P	***341.2183***	171.1128	324.1918	162.5995	3
7	802.4822	401.7447	785.4556	393.2314	P	***244.1656***	122.5864	227.1390	114.0731	2
8					K	***147.1128***	74.0600	130.0863	65.5468	1

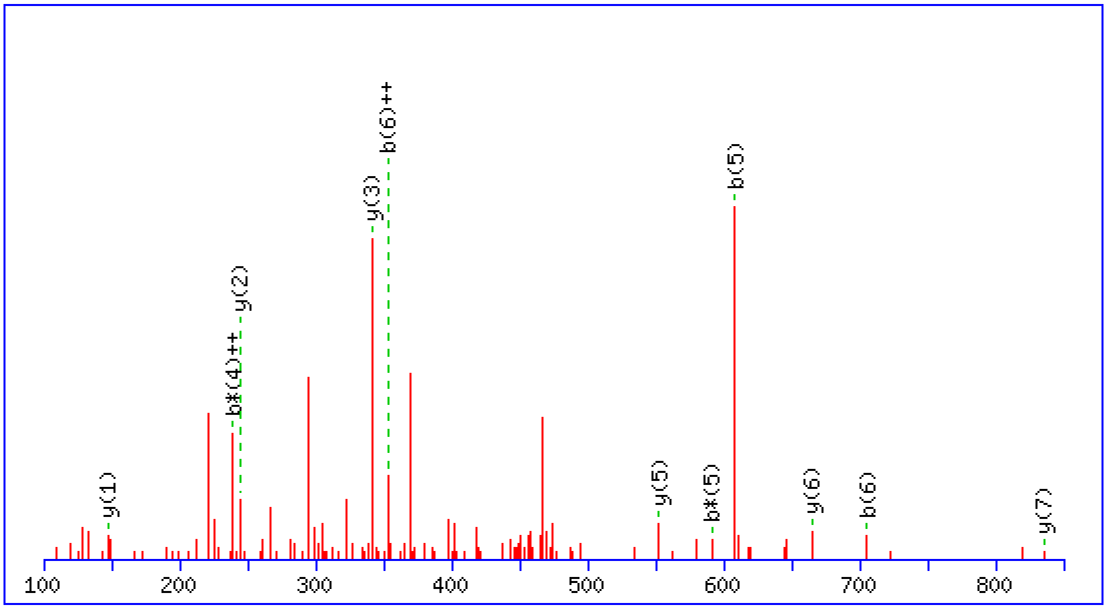

RUIL - Spot 2 - LipL32

Sequence: **QAIAAEESLKK**
MH+: 1228.654749
Charge: 3+
Ions Score: 42
Expect: 0.0001

#	b	b++	b*	b*++	b0	b0++	Seq.	y	y++	y*	y*++	y0	y0++	#
1	**129.0659**	65.0366	112.0393	56.5233			Q							11
2	**200.1030**	100.5551	183.0764	92.0418			A	1101.6514	551.3293	1084.6249	542.8161	1083.6408	542.3241	10
3	**313.1870**	157.0972	296.1605	148.5839			I	1030.6143	515.8108	1013.5877	507.2975	1012.6037	506.8055	9
4	384.2241	192.6157	367.1976	184.1024			A	917.5302	459.2687	900.5037	450.7555	899.5197	450.2635	8
5	455.2613	228.1343	438.2347	219.6210			A	846.4931	423.7502	829.4666	415.2369	828.4825	414.7449	7
6	584.3039	292.6556	567.2773	284.1423	566.2933	283.6503	E	775.4560	388.2316	758.4294	379.7184	757.4454	379.2264	6
7	713.3464	357.1769	696.3199	348.6636	695.3359	348.1716	E	**646.4134**	323.7103	629.3869	315.1971	628.4028	314.7051	5
8	800.3785	400.6929	783.3519	392.1796	782.3679	391.6876	S	517.3708	259.1890	500.3443	250.6758	499.3602	250.1838	4
9	913.4625	457.2349	896.4360	448.7216	895.4520	448.2296	L	**430.3388**	215.6730	413.3122	207.1598			3
10	1041.5575	521.2824	1024.5310	512.7691	1023.5469	512.2771	K	317.2547	159.1310	300.2282	150.6177			2
11							K	189.1598	95.0835	172.1332	86.5702			1

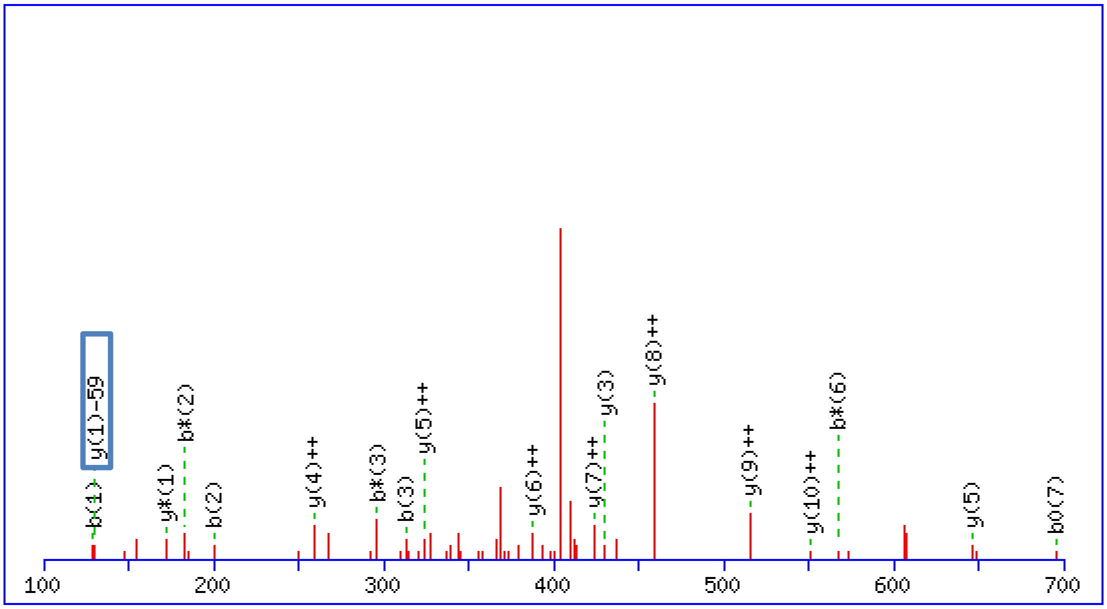

RUIL - Spot 2 - LipL32

Sequence: **QAIAAEESLKK**
MH+: 1228.666204
Charge: 2+
Ions Score: 84
Expect: 2.2e-007

#	b	b++	b*	b*++	b0	b0++	Seq.	y	y++	y*	y*++	y0	y0++	#
1	**129.0659**	65.0366	112.0393	56.5233			Q							11
2	**200.1030**	100.5551	183.0764	92.0418			A	***1101.6150***	551.3111	1084.5885	542.7979	1083.6045	542.3059	10
3	**313.1870**	157.0972	296.1605	148.5839			I	***1030.5779***	515.7926	1013.5514	507.2793	1012.5673	506.7873	9
4	**384.2241**	192.6157	367.1976	184.1024			A	***917.4938***	459.2506	900.4673	450.7373	899.4833	450.2453	8
5	**455.2613**	228.1343	438.2347	219.6210			A	***846.4567***	423.7320	829.4302	415.2187	828.4462	414.7267	7
6	584.3039	292.6556	567.2773	284.1423	566.2933	283.6503	E	***775.4196***	388.2134	758.3931	379.7002	757.4090	379.2082	6
7	**713.3464**	357.1769	696.3199	348.6636	695.3359	348.1716	E	***646.3770***	323.6921	629.3505	315.1789	628.3665	314.6869	5
8	800.3785	400.6929	783.3519	392.1796	782.3679	391.6876	S	***517.3344***	259.1709	500.3079	250.6576	499.3239	250.1656	4
9	913.4625	457.2349	896.4360	448.7216	895.4520	448.2296	L	***430.3024***	215.6548	413.2758	207.1416			3
10	**1083.5681**	542.2877	1066.5415	533.7744	1065.5575	533.2824	K	***317.2183***	159.1128	300.1918	150.5995			2
11							K	***147.1128***	74.0600	130.0863	65.5468			1

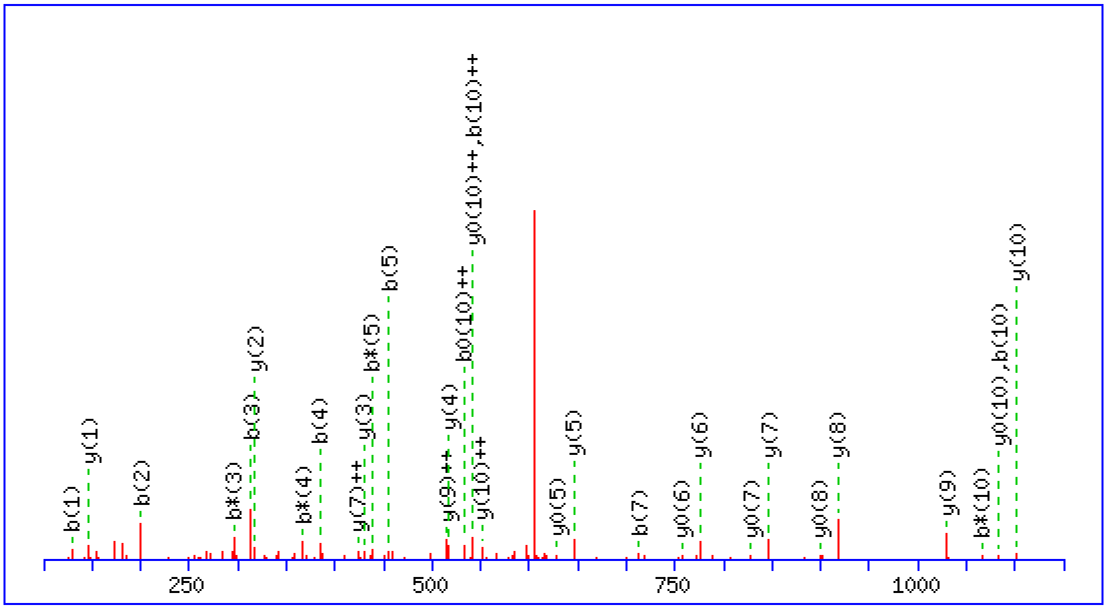

RUIL - Spot 2 - LipL32

Sequence: **SFDDLKNIDTK**
MH+: 1336.642326
Charge: 2+
Ions Score: 51
Expect: 0.00034

#	b	b++	b*	b*++	b0	b0++	Seq.	y	y++	y*	y*++	y0	y0++	#
1	88.0393	44.5233	70.0287	35.5180			S							11
2	235.1077	118.0575	217.0972	109.0522			F	1250.6627	625.8350	1233.6361	617.3217	1232.6521	616.8297	10
3	350.1347	175.5710	332.1241	166.5657			D	1103.5943	552.3008	1086.5677	543.7875	1085.5837	543.2955	9
4	465.1616	233.0844	447.1510	224.0792			D	988.5673	494.7873	971.5408	486.2740	970.5568	485.7820	8
5	578.2457	289.6265	560.2351	280.6212			L	873.5404	437.2738	856.5138	428.7606	855.5298	428.2686	7
6	748.3876	374.6974	731.3610	366.1842	730.3770	365.6921	K	760.4563	380.7318	743.4298	372.2185	742.4458	371.7265	6
7	862.4305	431.7189	845.4040	423.2056	844.4199	422.7136	N	590.3144	295.6608	573.2879	287.1476	572.3039	286.6556	5
8	975.5146	488.2609	958.4880	479.7476	957.5040	479.2556	I	476.2715	238.6394	459.2449	230.1261	458.2609	229.6341	4
9	1090.5415	545.7744	1073.5150	537.2611	1072.5310	536.7691	D	363.1874	182.0974	346.1609	173.5841	345.1769	173.0921	3
10	1191.5892	596.2982	1174.5626	587.7850	1173.5786	587.2930	T	248.1605	124.5839	231.1339	116.0706	230.1499	115.5786	2
11							K	147.1128	74.0600	130.0863	65.5468			1

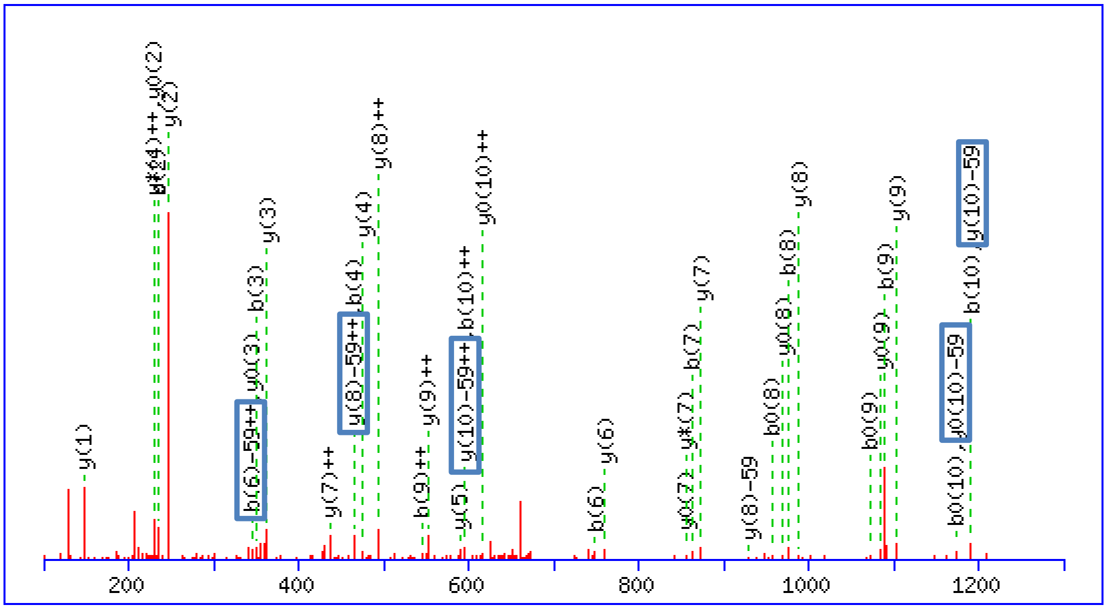

RUIL - Spot 2 - LipL32

Sequence: ISFTTYKPGEVK
MH+: 1410.731860
Charge: 2+
Ions Score: 68
Expect: 9.1e-006

#	b	b++	b*	b*++	b0	b0++	Seq.	y	y++	y*	y*++	y0	y0++	#
1	114.0913	57.5493					I							12
2	201.1234	101.0653	183.1128	92.0600			S	1298.6627	649.8350	1281.6361	641.3217	1280.6521	640.8297	11
3	348.1918	174.5995	330.1812	165.5942			F	1211.6307	606.3190	1194.6041	597.8057	1193.6201	597.3137	10
4	449.2395	225.1234	431.2289	216.1181			T	1064.5623	532.7848	1047.5357	524.2715	1046.5517	523.7795	9
5	550.2871	275.6472	532.2766	266.6419			T	963.5146	482.2609	946.4880	473.7477	945.5040	473.2556	8
6	713.3505	357.1789	695.3399	348.1736			Y	862.4669	431.7371	845.4403	423.2238	844.4563	422.7318	7
7	883.4560	442.2316	866.4294	433.7184	865.4454	433.2264	K	699.4036	350.2054	682.3770	341.6921	681.3930	341.2001	6
8	980.5088	490.7580	963.4822	482.2447	962.4982	481.7527	P	529.2980	265.1527	512.2715	256.6394	511.2875	256.1474	5
9	1037.5302	519.2688	1020.5037	510.7555	1019.5197	510.2635	G	432.2453	216.6263	415.2187	208.1130	414.2347	207.6210	4
10	1166.5728	583.7900	1149.5463	575.2768	1148.5623	574.7848	E	375.2238	188.1155	358.1973	179.6023	357.2132	179.1103	3
11	1265.6412	633.3243	1248.6147	624.8110	1247.6307	624.3190	V	246.1812	123.5942	229.1547	115.0810			2
12							K	147.1128	74.0600	130.0863	65.5468			1

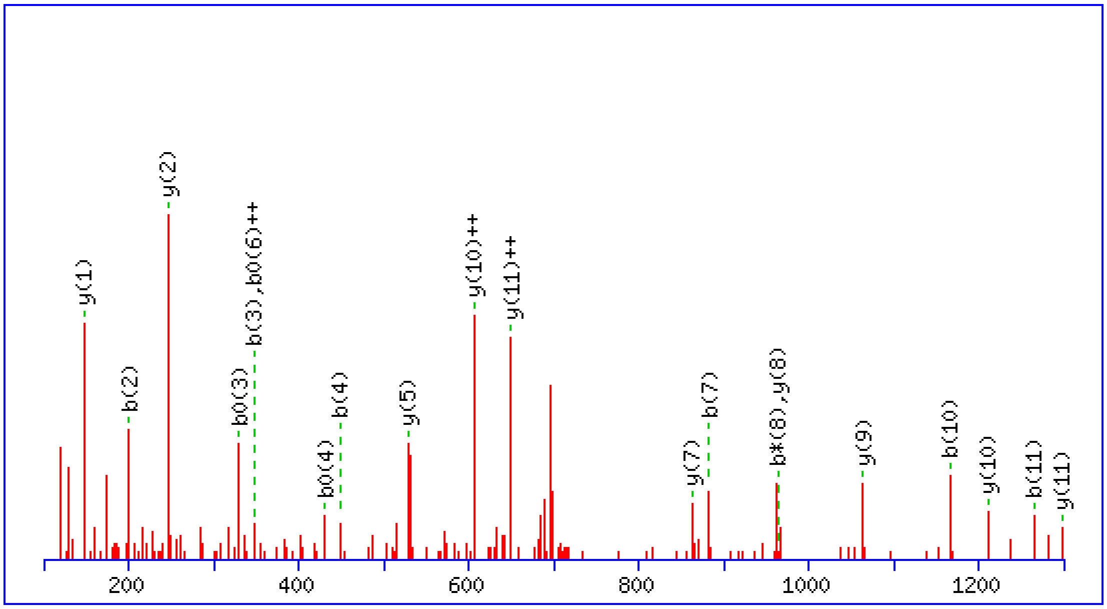

RUIL - Spot 2 - LipL32

Sequence: **LDDDDDGDDTYKEER**
MH+: 1841.709568
Charge: 2+
Ions Score: 48
Expect: 3.7e-005

#	b	b++	b*	b*++	b0	b0++	Seq.	y	y++	y*	y*++	y0	y0++	#
1	114.0913	57.5493					L							15
2	229.1183	115.0628	211.1077	106.0575			D	1729.6307	865.3190	1712.6042	856.8057	1711.6202	856.3137	14
3	344.1452	172.5763	326.1347	163.5710			D	1614.6038	807.8055	1597.5772	799.2923	1596.5932	798.8003	13
4	459.1722	230.0897	441.1616	221.0844			D	1499.5769	750.2921	1482.5503	741.7788	1481.5663	741.2868	12
5	574.1991	287.6032	556.1885	278.5979			D	1384.5499	692.7786	1367.5234	684.2653	1366.5393	683.7733	11
6	689.2261	345.1167	671.2155	336.1114			D	1269.5230	635.2651	1252.4964	626.7518	1251.5124	626.2598	10
7	746.2475	373.6274	728.2370	364.6221			G	1154.4960	577.7517	1137.4695	569.2384	1136.4855	568.7464	9
8	861.2745	431.1409	843.2639	422.1356			D	1097.4746	549.2409	1080.4480	540.7276	1079.4640	540.2356	8
9	**976.3014**	488.6543	958.2908	479.6491			D	982.4476	491.7274	965.4211	483.2142	964.4371	482.7222	7
10	1077.3491	539.1782	1059.3385	530.1729			T	867.4207	434.2140	850.3941	425.7007	849.4101	425.2087	6
11	1240.4124	620.7098	1222.4018	611.7046			Y	766.3730	383.6901	749.3464	375.1769	748.3624	374.6849	5
12	1410.5179	705.7626	1393.4914	697.2493	1392.5074	696.7573	K	603.3097	302.1585	586.2831	293.6452	585.2991	293.1532	4
13	1539.5605	770.2839	1522.5340	761.7706	1521.5500	761.2786	E	433.2041	217.1057	416.1776	208.5924	415.1936	208.1004	3
14	1668.6031	834.8052	1651.5766	826.2919	1650.5926	825.7999	E	**304.1615**	152.5844	287.1350	144.0711	286.1510	143.5791	2
15							R	**175.1190**	88.0631	158.0924	79.5498			1

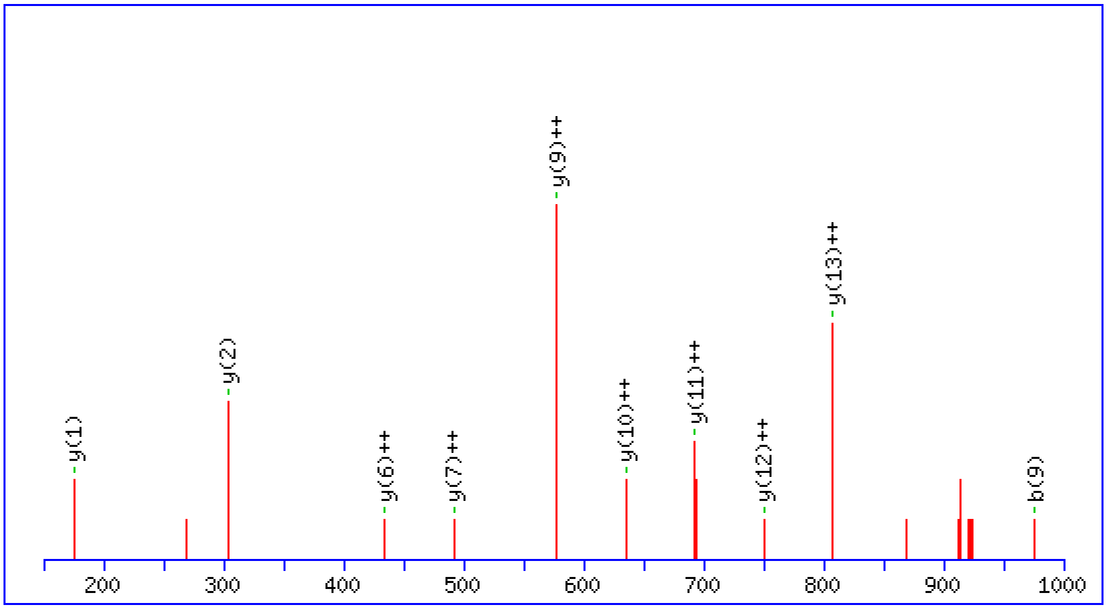

RUIL - Spot 2 - LipL32

Sequence: **SSFVLSEDTIPGTNETVK**
MH+: 1964.965992
Charge: 2+
Ions Score: 57
Expect: 1.9e-005

#	b	b++	b*	b*++	b0	b0++	Seq.	y	y++	y*	y*++	y0	y0++	#
1	88.0393	44.5233	70.0287	35.5180			S							18
2	175.0713	88.0393	157.0608	79.0340			S	1878.9331	939.9702	1861.9066	931.4569	1860.9226	930.9649	17
3	***322.1397***	161.5735	304.1292	152.5682			F	1791.9011	896.4542	1774.8745	887.9409	1773.8905	887.4489	16
4	***421.2082***	211.1077	403.1976	202.1024			V	1644.8327	822.9200	1627.8061	814.4067	1626.8221	813.9147	15
5	***534.2922***	267.6498	516.2817	258.6445			L	1545.7643	773.3858	1528.7377	764.8725	1527.7537	764.3805	14
6	***621.3243***	311.1658	603.3137	302.1605			S	***1432.6802***	716.8437	1415.6536	708.3305	1414.6696	707.8385	13
7	***750.3668***	375.6871	732.3563	366.6818			E	***1345.6482***	673.3277	1328.6216	664.8144	1327.6376	664.3224	12
8	865.3938	433.2005	847.3832	424.1952			D	***1216.6056***	608.8064	1199.5790	600.2932	1198.5950	599.8011	11
9	***966.4415***	483.7244	948.4309	474.7191			T	1101.5786	551.2930	1084.5521	542.7797	1083.5681	542.2877	10
10	1079.5255	540.2664	1061.5150	531.2611			I	***1000.5310***	500.7691	983.5044	492.2558	982.5204	491.7638	9
11	1176.5783	588.7928	1158.5677	579.7875			P	***887.4469***	444.2271	870.4203	435.7138	869.4363	435.2218	8
12	1233.5998	617.3035	1215.5892	608.2982			G	***790.3941***	395.7007	773.3676	387.1874	772.3836	386.6954	7
13	1334.6474	667.8274	1316.6369	658.8221			T	733.3727	367.1900	716.3461	358.6767	715.3621	358.1847	6
14	1448.6904	724.8488	1431.6638	716.3355	1430.6798	715.8435	N	***632.3250***	316.6661	615.2984	308.1529	614.3144	307.6608	5
15	1577.7330	789.3701	1560.7064	780.8568	1559.7224	780.3648	E	518.2821	259.6447	501.2555	251.1314	500.2715	250.6394	4
16	1678.7806	839.8940	1661.7541	831.3807	1660.7701	830.8887	T	389.2395	195.1234	372.2129	186.6101	371.2289	186.1181	3
17	1777.8491	889.4282	1760.8225	880.9149	1759.8385	880.4229	V	***288.1918***	144.5995	271.1652	136.0863			2
18							K	***189.1234***	95.0653	172.0968	86.5520			1

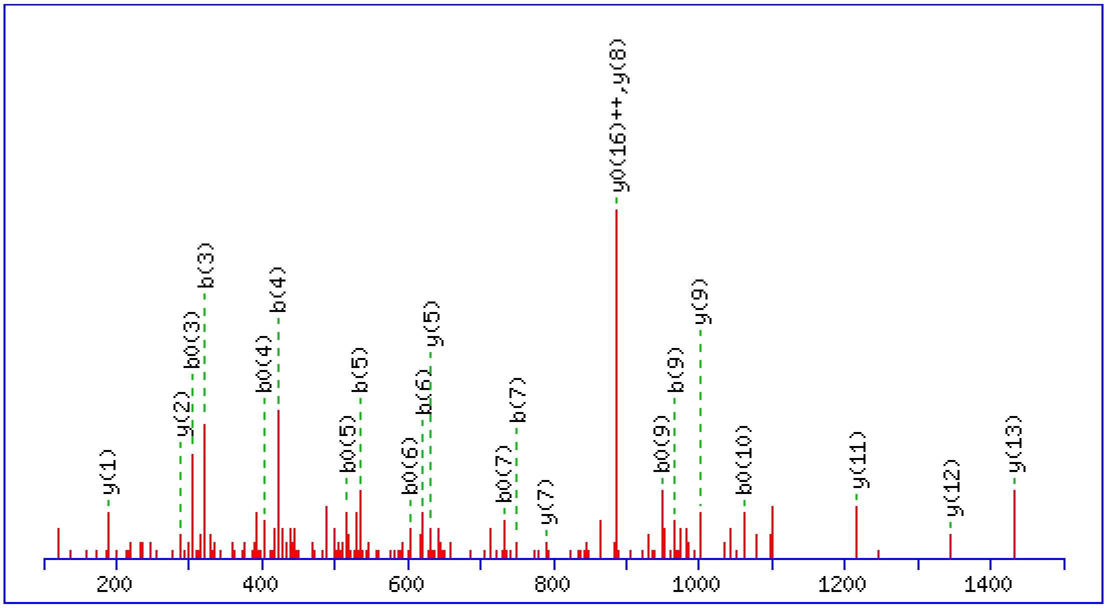

Supplement: Figure S1 — MS/MS spectra for peptides containing a modified lysine. Modified lysines are underlined in the peptide sequence. MH+-59 ions, which are neutral loss ions indicative of peptides containing a tri-methylated lysine [35], are indicated in blue boxes within the spectra. (PDF) [file pntd.0003280.s001.pdf]
